# Brain Iron Dyshomeostasis and Ferroptosis in Alzheimer’s Disease Pathophysiology: Two Faces of the Same Coin

**DOI:** 10.14336/AD.2024.0094

**Published:** 2025-06-10

**Authors:** Mariarosa Mezzanotte, Serena Stanga

**Affiliations:** Neuroscience Institute Cavalieri Ottolenghi, Department of Neuroscience Rita Levi Montalcini, University of Turin, 10126 Turin, Italy.

**Keywords:** dementia, iron overload, neurodegeneration, ferroptosis, mitochondria

## Abstract

Iron is a fundamental metal involved in many cellular and biological processes in all organisms, humans included. Iron homeostasis is finely regulated both systemically and at the level of the Central Nervous System (CNS) to avoid its imbalance; indeed, iron excess is extremely toxic for cells and triggers detrimental oxidative stress increase. Nevertheless, factors such as genetics, environment, and aging can alter the normal iron metabolism leading to diseases, including neurodegenerative disorders such as Alzheimer's disease (AD). AD is the most widespread neurodegenerative disorder of the CNS. Although the precise pathogenesis of AD is not clarified yet, different studies conducted both in mouse models and in patients reported brain iron accumulation, resulting in cognitive, memory, and motor decline. Moreover, the expression of many proteins involved in iron metabolism appears to be altered in the brain, leading to iron deposition and promoting AD progression. In the context of AD, amyloid beta (Aβ) and tau hyperphosphorylation, the two hallmarks of the disease, can promote brain iron deposition and subsequent neuronal death. Indeed, although the mechanism of neuronal loss is not fully understood, several evidence demonstrated the involvement of the iron-dependent form of cell death defined as “ferroptosis”. In this review, we deepened about the role of iron and iron deregulation in the CNS with a particular focus on its involvement in the pathogenesis of AD. We also discussed the potential role of ferroptosis as a new pathological mechanism related to dementia. Finally, we reviewed recent strategies for treating AD based on the use of iron chelators, antioxidants and ferroptosis inhibitors, paying close attention to iron disorders and the development of new drugs capable of preventing AD.

## 1. Introduction

The relevance of iron to nearly all living organisms is indisputable. Iron is the most physiologically copious transition metal, and it is required in various fundamental biological processes essential for life. Indeed, iron is critical to physiological cellular homeostasis since it works as a cofactor for proteins involved in essential (ATP production, DNA biosynthesis/repair, cell division) and specialized (oxygen transport, neurotransmission) cellular functions. A commonly shared property among transition metals, including iron, is their ability of interconversion between the divalent cation or ferrous (Fe^2+^) and trivalent cation or ferric (Fe^3+^) states [1]. The ability to accept and donate electrons makes iron an essential component of oxygen-binding molecules (Hemoglobin and Myoglobin), cytochrome, in the electron transport chain and as a cofactor in a variety of enzymes. However, this property turns iron also into an extremely potentially harmful metal. Indeed, under aerobic conditions iron can readily catalyze the generation of toxic radicals through the Fenton reaction, in which hydrogen peroxide (H_2_O_2_) is converted into the highly reactive hydroxyl radical (·OH), which can damage membrane lipids, proteins, and DNA, and cause cell death and tissue damage [2]. This Janus-faced effect is related to disorders characterized by either iron excess or deficiency and altered distribution.

Iron balance is controlled on the one hand by dietary uptake and absorption, on the other hand by iron release from recycling macrophages and hepatocytes; however, the physiological mechanism of iron excretion is not regulated [3]. Therefore, iron can be deposited in specific tissues with age.

In the aging brain, iron accumulates in regions which are involved in neurodegenerative disorders, such as the cerebral cortex, hippocampus, and striatum [4-6]. In the Central Nervous System (CNS), the maintenance of balanced iron levels is crucial, since its dyshomeostasis leads to oxidative stress, inflammatory response with consequent damage, cell death, and, finally, neurological diseases [7]. Recently, it has been described a new type of iron-dependent cell death caused by iron overload: “ferroptosis” [8]. This process could be a new plausible mechanism inducing neuronal death in neurodegenerative diseases, such as Alzheimer’s disease (AD) [9].

In this review, we discuss the mechanisms of iron regulation/deregulation during healthy aging and neurodegeneration, with a particular focus on AD. Moreover, the hypothesis of ferroptosis driving neurodegeneration and the targeting of ferroptosis as a potential therapeutic target, are elaborated.

## 2. Iron metabolism

### 2.1 Iron absorption, storage and excretion

Iron intake, in the form of heme and non-heme iron, occurs through the diet. Only 1-2 mg of iron is assimilated daily in the gut and the same amount is lost in the urine, feces, sweat and sloughed cells. Macrophages store iron and recycle it from the destruction of senescent red blood cells. The reuse of iron by macrophages is essential in the process of erythropoiesis as it provides approximately 20-25 mg of iron per day [10]. Heme iron is introduced into the enterocytes via a Heme carrier protein 1 (HCP1), a heme receptor localized on the brush border of intestinal cells. Heme is broken up into free iron and biliverdin by heme oxygenase (HMOX). Once released, iron then enters the low-molecular weight pool and is transferred outside from the enterocyte in the same way as inorganic non-heme iron. HCP1 and the major transport facilitator for feline leukemia virus, subgroup C (FLVCR), have been shown to export cytoplasmic heme in human erythroid cells, suggesting that intact heme may also be conducted out of the enterocyte [11]. Inorganic iron is absorbed by duodenal enterocytes via the Divalent Metal Transporter 1 (DMT1) [12] after reduction of iron from ferric (Fe^3+^) to ferrous (Fe^2+^) by duodenal cytochrome b (DcytB) localized in the apical membrane of enterocytes [13]. The fundamental role of DMT1 in intestinal iron intake has been proven by using animal models with intestine deletion of DMT1 that provoke postnatal anemia and systemic iron reduction [14].

Depending on the body needs, in enterocytes, non-used iron can be kept by ferritin, a major iron storage protein, or exported to bloodstream by Ferroportin1 (Fpn1), the only iron exporter [15]. The exported ferrous iron is then oxidated to ferric iron by Hephaestin (HEPH), a membrane ferroperoxidase [16] and binds to circulating plasma Transferrin (Tf) [17].

Holo-transferrin (Tf-Fe^2+^) binds to Transferrin Receptor 1 (TfR1) on the cell surface and is internalized through a receptor-mediated endocytosis. Iron in the endosome is released from Tf and reduced by metalloreductase, Six transmembrane epithelial antigen of the prostate 3 (Steap3) [18]. After reduction, Fe^2+^ is transported into the cytosol by DMT1 or ZRT, IRT-like protein (ZIP14) [19]. Both apo-Tf and TfR1 return to the cell surface, where the iron-depleted Tf is released allowing TfR1 to bind other iron-loaded Tf for another round of internalization.

### 2.2 Systemic iron homeostasis: Hepcidin-Ferroportin1 (Hepc-Fpn1) axis

The key regulator of systemic iron homeostasis is Hepcidin (Hepc), a small peptide of 25 amino acids, secreted by liver hepatocytes. Hepc secretion is regulated at the transcriptional level by different stimuli including systemic iron availability, liver iron stores, hypoxia, erythropoietic activity, and inflammatory/infectious states [20]. Physiologically, in iron overload conditions Hepc is upregulated, displaying a regulatory response to iron overload, while during iron deficiency condition the Hepc synthesis is reduced. Hepc regulates iron export to the plasma via lysosomal degradation of the iron exporter Ferroportin1 (Fpn1) in enterocytes, macrophages and hepatocytes [21]. The mechanism of action of Hepc-Fpn1 pathway implies that when Hepc levels are low (iron-deficiency states), Fpn1 levels and iron release by macrophages and duodenal crypt cells are up-regulated. In contrast, when Hepc levels are high, Fpn1 is downregulated resulting in iron retention within these cells.

Different hemochromatosis proteins are involved in Hepc regulation: hereditary hemochromatosis protein (HFE), Transferrin receptor type 1 and 2 (TfR1 and TfR2), Hemojuvelin (Hjv), bone morphogenetic protein (BMP). These proteins coordinate the SMAD pathway signaling through the binding of Bone morphogenetic proteins to their receptors [22].

In particular, Bone morphogenetic protein 2 (BMP2), BMP6 and two types of BMP receptors, type I (BMPRI) and type II (BMPRII) are involved in this pathway activation. BMP2 and BMP6, the iron dependent proteins, work as a heterodimer activating Hepc *in vivo* [23].

When iron levels are high, the BMP6 expression increases and binds to BMP receptors, BMPR1-2, on the surface of hepatocytes in presence of the co-receptor Hemojuvelin (HJV). BMP6 activates the signal transduction through SMAD1/5/8 phosphorylation (pSMADs), the formation of a complex with SMAD4 (pSMADs/SMAD4) that translocates into the nucleus where it activates HEPC gene transcription [24]. On the other hand, in conditions of low iron levels, Hepc expression is inhibited. Transmembrane serine protease matriptase 2, codified by TMPRSS6 gene [25] is the key protein involved in this mechanism. It downregulates BMP/SMAD signaling to Hepc since it cleaves and forms a soluble form of Hjv, one of the Hepc positive regulators, inactivating it [26].

Serum iron level can stimulate Hepc in a BMP6 independent way that involves saturated Tf, marker of increased iron availability. HFE, TfR1 and TfR2 are involved in this signal transduction too.

When Transferrin saturation increases, the SMAD 1/5/8 phosphorylation is induced by a mechanism involving HFE protein [27]. Since HFE competes with Tf for binding to TfR1, when circulating holo-transferrin raises, HFE dissociates from TfR1 and it is able to interacts with TfR2 and HJV in order to induce BMP-SMAD signaling to Hepc [28].

In addition to iron, Hepc expression is induced by inflammatory/infection state. Indeed, interleukin-6 (IL6)/Janus Kinase 2 (JAK2) pathway is the main pathway that takes place in induction of Hepc promoter in inflammatory conditions [24]. Moreover, Hepc is controlled by negative regulators: Erythroferrone (ERFE) and Platelet-derived growth factor-BB (PDGF-BB) are candidate factors of Hepc inhibition exerted when erythropoiesis is compromised or in hypoxic conditions, respectively [29-31]. Focusing on ERFE, which is produced by erythroblasts in response to erythropoietin, it has been demonstrated that its deletion in an animal model of thalassemia intermedia (Th3/+) restored Hepc levels and, as consequence, iron levels in the liver and spleen of Th3/+ animals [31].

### 2.3 Intracellular iron metabolism: the IRE/IRP system

Cellular iron homeostasis is highly regulated by post-transcriptional mechanisms which control the expression of proteins involved in iron intake, release and storage, which are mainly regulated by the iron response element-iron regulatory protein (IRE-IRP) system. Several mRNAs that regulate iron homeostasis contain iron-responsive elements (IREs), a stem-loop structures located at the 5’- or 3’- untranslated regions (UTRs) flanking their coding sequence (CDS). IRE elements are bound by two functionally similar iron regulatory proteins, IRP1 and IRP2 [32]. Depending upon whether the IRE elements are located in the 5’-UTR or in the 3’-UTR, and if the levels of iron are low or high, the IRE-IRP interaction has opposite effects on the target gene expression. IRE/IRP complexes within the 5′UTR of an mRNA e.g. Ferritin H and L (Ft-H and L), Aconitase 2 (ACO2), FPN1 and Amyloid Precursor Protein (APP) inhibit translation, whereas IRP binding to IREs in the 3′UTR of TFR1 mRNA prevents its degradation [29].

In low iron states, IRP1 and 2 increase iron uptakes by stabilizing TfR1 mRNA and blocking iron storage and export by suppressing Ferritin and Fpn1. This homeostatic response mediates raised cellular iron intake from Tf and prevents the formation of Ferritin, useless in iron deficiency. On the contrary, in condition of iron overload, the lack of bind IRP-IREs induces iron storage (Ferritin) and export (FPN1) [33].

## 3. Brain Iron

The brain presents several peculiarities that make it unique, also regarding iron metabolism. Indeed, brain iron is involved in a variety of neurological processes such as myelination of axons, neuronal cells division and dopaminergic neurotransmitters synthesis, especially in the synthesis and signaling of monoamines [34]. It acts as a cofactor for proteins such as phenylalanine hydroxylase, tyrosine hydroxylase, and tryptophan hydroxylase [35]. Iron is present in different cells types and areas, from neurons to astrocytes and oligodendrocytes, in the interstitial space, in the soma and in the processes of nerve cells [36]. As in peripheral organs, increased brain iron levels work as a potent neurotoxin; indeed, iron produces toxic radicals which cause damage [37] and can lead to Neuro Degenerative Diseases (NDDs) [36]. The brain, and in general the CNS, is highly susceptible to free radicals for two main reasons: i) the brain is not particularly rich in antioxidants, ii) it uses high oxygen levels and it contains a high concentration of oxidizable polyunsaturated fatty acids [38].

### 3.1 Iron import/transport across the BBB

The blood-brain barrier (BBB) represents an efficient protective filter for the brain, which protects it against the passage of potentially harmful molecules. The BBB separates also brain iron homeostasis from the systemic one. Indeed, in condition of systemic iron accumulation, like hemochromatosis, the cellular damage related to iron is not reflected at the level of the CNS [39], since BBB regulates iron entry inside the brain.

Iron uptake in the brain occurs through Transferrin Receptor 1 (TfR1) that is expressed on the luminal side of brain capillaries. TfR1 binds circulating transferrin (Tf-Fe^2^) to favor iron internalization into brain microvascular endothelial cells (BMVECs) through TfR1-mediated endocytosis mechanism [6]. Iron is released into the cytoplasmic space and exported at the abluminal membrane level by unknown pathways that could involve Fpn1 [40]. Fpn1 can export Fe^2+^ out of the capillary endothelial cells where, by the action of Cerulosplamin (CP), Fe^2+^ is oxidized to Fe^3+^ [41]. Into intracellular and cerebrospinal fluids, the Tf-Fe complex can bind TfR1 receptor on the surface of nerve cell membranes, as consequence iron is released and the resulting apo-TF can enter into the blood circulation through arachnoid villi [36]. Also in the brain Fpn1 is regulated by the Hepc expressed in mature astrocytes, oligodendrocyte [42] and neurons both in human [43] and in mouse [44], where it covers a role in controlling of iron amount [45]. Recently, we and other demonstrated that there is an endogenous cerebral Hepc expression and that it is regulated by the levels of iron in the brain itself [4-6, 46]. However, it could also be that the brain Hepc comes from liver [42] since the peptide size [47] and its amphipathic cationic chemical structure [48] would allow it to cross the BBB.

**Figure 1.**
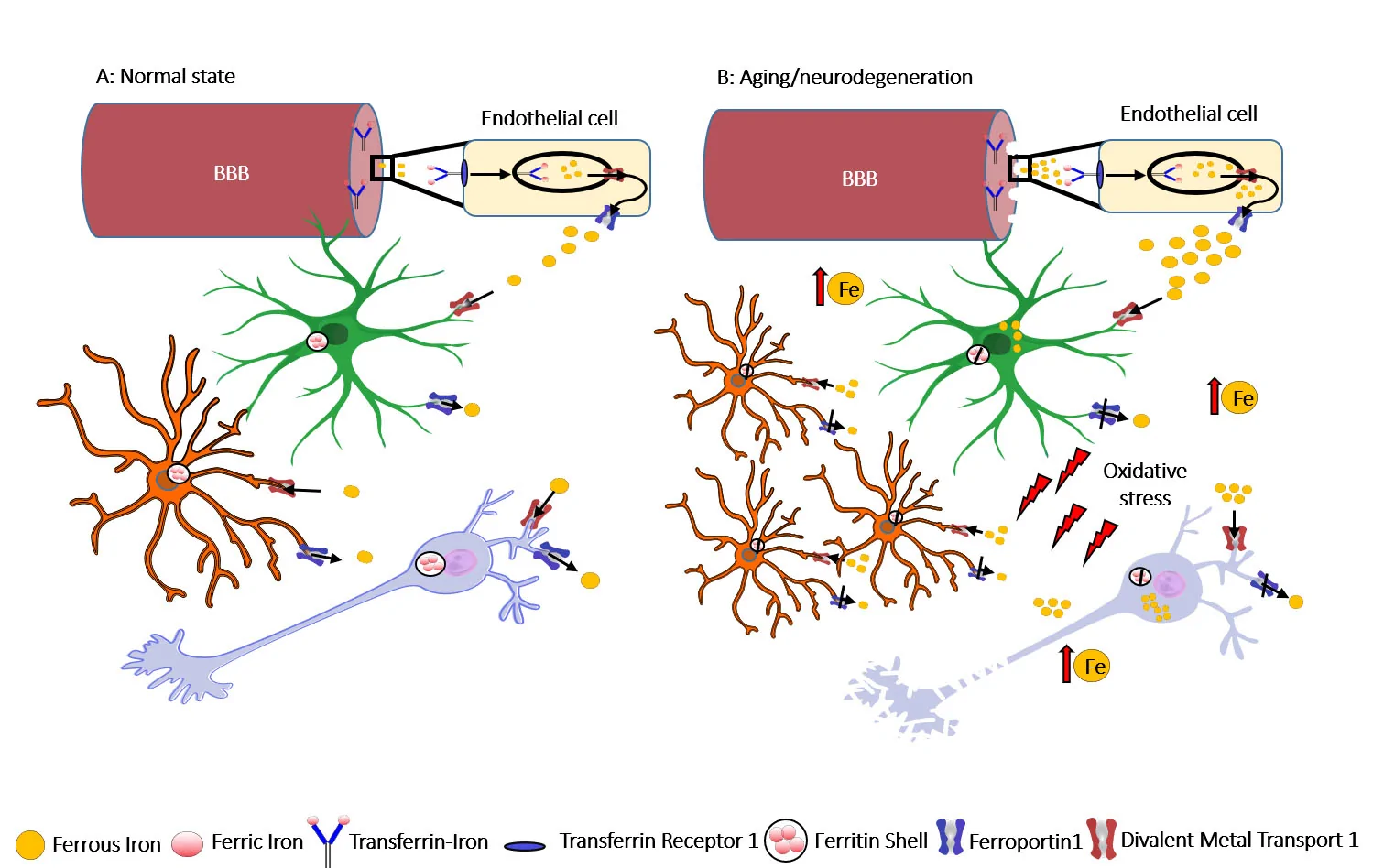
**Iron homeostasis and dyshomeostasis**. (**A**) Normal state: circulating iron can cross the BBB through its binding to transferrin and through the mechanism of endocytosis it is absorbed by endothelial cells that express the Transferrin Receptor 1 (TfR1). Once in the brain, iron is absorbed by Divalent metal transporter 1 (DMT1) and continually moves between astrocytes, neurons, oligodendrocytes, and microglia. The mechanism of iron uptake in neurons and microglia is mediated via transferrin receptors. Ferric iron can be stored in a safe way inside the ferritin shell or can be exported from cells involved in the iron exporter, Ferroportin1 (Fpn1), present in all cell types. (**B**) Aging and neurodegeneration: during aging and neurodegeneration the BBB damage can induce an increase of iron entry with a consequence alteration of iron metabolism in the brain with the accumulation of redox-active ferrous iron in all brain cells. This is accompanied by microglia activation, astrogliosis and neuron degeneration. This could be attributed to alterations both at the level of expression and functions of important regulatory proteins such as Ferritin and Ferroportin1, which, being unable to export iron from cells, increases its accumulation and consequently the labile iron content. At the microglial level, it can cause a release of pro-inflammatory Cytokines with an increase of microglia activation. Overall, brain iron deposition can promote oxidative stress that induces neurodegeneration. Color legend: Astrocytes in green; Microglia in orange and neurons in blue. Inkscape software (Free vector graphics editors) was used to create the image.

### 3.2 Brain iron accumulation

Brain iron homeostasis should be carefully protected to avoid neurotoxicity. Despite this, conditions closely related to aging, such as inflammation and BBB damage [49], can be the cause of misdistribution and imbalance of iron in this organ [50, 51]. Therefore, the process of aging is a suitable model to study iron metabolism alteration also found in NDDs [52]. Indeed, aging is the leading cause of neurodegeneration and aged-dependent brain iron accumulation could result from a change in iron proteins levels that impair its homeostasis (Fig. 1). In this context, various researchers have confirmed that iron, Transferrin and Ferritin levels are altered during aging in human astrocytes and oligodendrocytes [53].

Ferritin, the “saving protein” is successfully related to iron overload: if on one hand, under conditions of cellular iron demand, ferritins can release iron to be used for metabolic processes, on the other hand it can store iron in the cell to reduce cell damage and oxidative stress [54]. In humans three isoforms of ferritin are present encoded by three different genes: FTL gene which encodes the cytosolic Light chain (FtL), FTH gene coding for the cytosolic Heavy chain (FtH) and the intron-free gene FTMT involved in the encoding of mitochondrial ferritin (FtMt) [55]. Cytosolic ferritin composition is made of 24 chains of Heavy (H), with ferroxidase activity, and Light (L) isoforms able to facilitate iron hydrolysis and mineralization [56]. The two chains co-assemble to form heteropolymers with a specific ratio depending on the organ/tissue type [57]. It has been demonstrated that there is a quite high level of FtH chain compared to FtL chain in younger individuals, while ferritins heteropolymers increase with age in the frontal cortex, caudate, putamen, substantia nigra and globus pallidus [58]. The same ratio were observed in rats’ brains [59]. Instead, the mitochondrial ferritin subunit precursors are channeled inside the mitochondria, where after cleavage of the N-terminal leader sequence, the mature subunits assemble to form a homopolymers composed by 24 subunits of FtMt [60]. In spite of this difference, from a functional and structural point of view, mitochondrial ferritin is similar to cytosolic ferritin; once iron is internalized in mitochondria, it can be stored in mitochondrial ferritin (FtMt), to avoid ROS production through Fenton reaction [61, 62]. FtMt expression depends on the tissue, and it is mostly expressed in testis and brain [61, 63]. FtMt homopolymers can incorporate mitochondrial iron. The increase of FtMt determines a passage of iron from cytosol to the mitochondria that induces mitochondrial iron accumulation with a consequent reduction of cytosolic iron availability [61]. Therefore, FtMt increased expression is indicative of disruption of cellular iron homeostasis, but at the same time it suggests that FtMt can cover an important role in controlling systemic iron levels.

### 3.3 Iron metabolism in the nervous tissue

The mechanism of iron uptake in the brain follows the same route as the systemic one. The exact mechanism of iron import from blood to brain parenchyma via Blood Brain Barrier (BBB) is not fully understood but there are two plausible hypotheses. The first predicts that iron can be transported into the brain interstitium through a process of receptor-mediated transcytosis of iron-loaded transferrin (holo-Tf). In this model holo-Tf can cross the cytosol of brain capillary endothelial cells (BCECs) of the BBB to be directly released into the brain [64]. The second hypothesis provides the endocytosis mechanism of the holo-Tf-TfR1 complex. After endosome acidification, iron is dissociated from Tf and only after its reduction in Fe^2+^, it can be transfer from endosomal membrane into the cytosol via DMT1 [65] which will be used for normal neuronal metabolism and partly stored in ferritin in order to protects neurons to excess of iron [66]. In condition of neuronal iron deficiency, the lysosomal ferritin degradation allows the release of iron to compensate its deficiency to the physiological needs of the neurons [67, 68].

The iron export from neurons is mediated by Fpn1 through Fpn1/Hephaestin (Heph) and Fpn1/ Ceruloplasmin (Cp) pathway [69, 70]. It has been demonstrated that alterations in this pathway can induce iron retention and, consequently, memory damage [71, 72].

Neuronal iron homeostasis is maintained and supported by glial cells, astrocytes and microglia. Both astrocytes and microglia express TfR1 and DMT1 which mediate iron influx from BMECs and the brain interstitium controlling iron amount in neurons [70, 73]. In particular, astrocytes are able to resist metal-induced toxicity [74], and they act as a support for the maintenance of optimal neuronal functions [75]. However, even though the astrocytes are more resistant to iron toxicity than neurons and oligodendrocytes, in an environment with high iron levels, the increase of glial fibrillary acidic protein (GFAP) was found [74]. In condition of iron overload, astrocytes activation induces the release antioxidant factors [76] and inflammatory mediators, that for example, in the context of AD can induced an oxidative state which enhances the Aβ and tau formation [77]. Moreover, in condition of iron deficiency, astrocytes and microglia are able to release iron bound to ferritin and to support oligodendrocytes, where TfR1 is absent [78], in the myelination processes [79]. On the other hand, in condition of iron overload the oligodendrocytes, can overexpress ferritin providing an important antioxidant defense for neurons [80].

Microglia is the major iron-responsive cell in the CNS; in condition of iron overload microglia is activated [81] with evident increase of the soma volume and the reduction of the processes’ length [82]. Microglia activation is induced by iron through the release of proinflammatory cytokines mediated by the nuclear factor-κB (NF-κB) [81], which by induction of ferritin-light chain (FtL) upregulation, causes iron retention. This mechanism has been observed also in AD, where in condition of iron overload, microglia is activated and is able to sequester iron [83].

Interestingly, microglia activation can promote also the release of lactoferrin (Lf) which is able to bind APP [84] promoting the increase of IL-1β expression in microglia in condition of iron overload, intensifying the proinflammatory effects [85].

Exposure of microglia to interferon (IFN) γ and Aβ promote an inflammatory phenotype accompanied by iron retention and reduction of both phagocytic and chemotactic ability. In the same study, microglia isolated from APP/PS1 mice also show an iron-retentive phenotype with a reduction of Aβ phagocytosis [86].

### 3.4 Iron accumulation within the cell: focus on the role of mitochondria

Nervous tissue is particularly rich in mitochondria, fundamental organelles for the production of energy, devoted to neuronal communication/circuits. Indeed, the normal physiological processes of the cells in general and of the nervous tissue, in particular, depend on mitochondrial functions.

Interestingly, iron is an essential element for the maintenance of the latter, and an incorrect functioning of iron metabolism in mitochondria can lead to disease [87]. Mitochondria are the main organelles in which iron is used and accumulated. In mitochondria, iron is needed i) for heme synthesis and ii) Fe-S cluster biogenesis [88], iii) as essential cofactor for enzymes involved in the respiratory chain and Tricarboxylic Acid Cycle (TCA), or iv) it is exported by the heme-carrier feline leukemia virus subgroup C receptor 1 (FVLCR1b) and ISC-carrier ATP binding cassette subfamily B member 7 (ABCB7) for the activity of cytosolic enzymes [89].

The mechanism of iron uptake in the mitochondria is still controversial and a possible hypothesis has been proposed. One of these if the “kiss and run” model, studied in erythroid cells, in which iron (Fe^2+^) bypass the cytosol and after the fusion of transferrin (Tf)-endosomes with the mitochondrial membranes, iron can be internalized [90]. Authors demonstrated by time lapse confocal microscopy the co-localization of Tf vesicles and mitochondria [90].

Another study suggests that, after Fe^2+^ release by endosomes, iron is complexed to cytosolic chaperones which act as iron chelators, until the complex is transported to mitochondria. This theory came from Shvartsman and colleagues, who studied mouse cardiomyocytes treated with non-Tf-bound iron showing, through the use of specific iron chelators, that mitochondrial iron absorption is not obstructed [91]. It cannot be excluded that the mitochondrial iron release mechanisms are specific to the cell type. Related to this, it was be demonstrated that Transferrin receptor 2, the homologous of TfR1 [92], mainly expressed in hepatocytes, it is also expressed in nigral dopamine neurons. Interestingly, the complex transferrin/transferrin receptor 2 (Tf/TfR2), mediates iron import inside mitochondria of nigral dopamine neurons [93]. This because it has been identified in the TfR2 gene a mitochondrial targeting sequence that explained the TfR2 localization in both the plasma membrane and mitochondrial inner membrane of nigral dopamine neurons *in vivo*.

Furthermore, *in vitro* experiments demonstrated that TfR2 can deliver iron into the mitochondria since the lack of TfR2 reduced iron uptake into these organelles [93].

Noteworthy is the presence of transporters, mitoferrins (Mfrn 1/2), located in the inner mitochondrial membrane of mammalian cells which transport iron (Fe^2+^) from the intermembrane space to the matrix. While the Mfrn 1 is more expressed in hematopoietic tissues such as bone marrow, spleen, and liver on the other hand the expression of Mfrn 2 appears to be ubiquitous [94]. However, it has been shown that overexpression of both Mfrn 1/2 does not lead to an increase in mitochondrial iron content assuming that regulatory mechanisms may bypass mitoferrins to avoid excessive mitochondrial iron content [95]. Both regulatory mechanisms and the proteins involved need further investigation. Once it has penetrated into the matrix, iron can be used for the iron-sulfur cluster and heme synthesis, or it can be stored in the mitochondria. It is interesting to note that mitochondria, following the loss of electrons during oxidative phosphorylation, constitutes a cellular site of ROS production which can damage key lipids, proteins, and nucleic acids. In addition, since the mitochondria require a constant influx of iron for the iron-sulfur (Fe-S) cluster and heme synthesis, it is possible that further ROS are produced through the Fenton reaction. Mitochondria carry out two strategies to avoid oxidative stress: they can either use iron directly or they can incorporate it into the main storage protein, mitochondrial ferritin (FtMt) [60]. As described in the paragraph 3.2, the nuclear coding sequence of this protein is quite similar to that of the cytosolic form of ferritin, FtH. As well as FtH, FtMt oligomerizes to form a shell with ferroxidase activity involved in the oxidation of iron (from Fe^2+^ to Fe^3+^) and in order to sequester iron from reactive molecules within the matrix [60].

Moreover, mitochondria are dynamic organelles in constant transition between fission and fusion processes at the level of the outer (OMM) and inner (IMM) mitochondria membranes. Dynamin-Related Protein 1 (DRP1) allows mitochondria to divide and generate small mitochondria (fission); while they undergo fusion forming a large mitochondrion mediated by Mitofusin 1 and 2 (MFN 1 and 2), located in the outer mitochondrial membrane and the dynamin-like GTPase protein Optic Atrophy 1 (OPA1), localized to the intermembrane space [96]. These processes are important to maintain both mitochondria functionality and morphology [97].

In cells, in physiological conditions, a balance between fission and fusion can be compromised in different pathologies including NDDs [98, 99]. Related to neurodegenerative disease, it is known that iron accumulation occurs in caudate nucleus, putamen, nucleus accumbens, globus pallidus, and substantia nigra, located within the brain and while the transport of iron into the mitochondrion is increased, its storage and release are limited. This inevitably causes an increase in the production of ROS in neurons accompanied by a reduction in the synthesis of both heme and Fe-S clusters, leading to neuronal death and therefore neurodegenerative diseases [100].

Indeed, since iron covers a predominant role in mitochondrial oxidative functions, the imbalance of fission and fusion is linked to dyshomeostasis of iron. In particular, in iron overload condition ROS production can stimulate the process of fission by upregulating DRP1 [101, 102]. In mouse hippocampal neurons, it was shown that mitochondrial fission can be induced by iron overload, and it is associated with calcium signaling, which in turns can regulate DRP1 activity.

In particular, iron levels can accelerate the activity of calcineurin with a consequent increase of intracellular calcium levels that can enhance mitochondrial fission process and influence neurodegeneration [103]. Moreover, the increase in Fe^3+^ provokes the reduction of OPA1 expression causing an increased mitochondrial fragmentation [104-106]. Within neurons, mitochondrial concentration is higher in presynaptic regions and the ability of mitochondria to modulate Ca^2+^ flux is fundamental for the release of neurotransmitters, neurogenesis, and neuronal plasticity [107]. Neurons depend on mitochondrial oxidative phosphorylation (OXPHOS) to cover their energy demands. Interestingly, a recent work demonstrated that when young and healthy liver mitochondria are injected in the hippocampus of aged mice, where it is know that iron level are high [4], there is the improvement of mitochondrial functions through the upregulation of the mitochondrial complex II protein subunit SDHB demonstrating a key role of mitochondrial complex II in the aging process [108].

Mitochondrial dysfunctions with the consequent energy failure and production of reactive oxygen species (ROS), are two events inducing neurons loss in brain disease [97, 107, 109]. Data in literature demonstrate that increased mitochondrial iron plays a key role in both the initiation and progression of NDDs [110]. Indeed, mitochondrial bioenergetics is on the one hand directly influenced by the deregulation of iron through the inhibition of the electron transport chain (ETC) while on the other it distributes through the increase of the generation of ROS. In NDDs mitochondrial dysfunction is detected by increased ROS content, calcium and lipid peroxidation such as by a morphological alteration of the mitochondria [111].

As widely debated, mitochondria have a key role in the regulation of cellular iron metabolism, and they appear to be compromised in neurodegenerative diseases. This increases the possibility that their dysfunction may induce iron dyshomeostasis causing further mitochondrial dysfunction.

## 4. Iron dyshomeostasis: a new pathogenic mechanism in Alzheimer’s disease?

AD is the most widespread age-related neurodegenerative disorder that induces to dementia, characterized by impairment in cognitive, learning functions and memory loss [112, 113]. The majority of AD cases are sporadic, and aging is considered the most important risk factor; but genetically speaking AD is divided into familial and sporadic cases with a strong prevalence in female. If on the one hand the familial form of AD is caused by mutations in three important genes APP, Presenilin1 and 2 (PSEN1 and PSEN2) gene, on the other hand in the sporadic form of AD both hereditary and environmental factors can contribute to its onset [114, 115]. The main pathological features of AD in the brain are intracellular fibrillary tangles (NFTs) formed by hyperphosphorylated tau which lead to hippocampal and cortical neurons’ death and the extracellular accumulation of senile plaques derived from the aggregation of amyloid beta peptide (Aβ), the proteolytic product of APP [113].

Tau is a microtubule associated protein codified by the MAPT gene located on chromosome 17 [116]. Mutations in this gene are responsible for the familial form of tauopathies (*e.g* Frontotemporal dementia and parkinsonism linked to chromosome 17, FTDP-17)[117], while the causes for the sporadic form (*e.g AD)* are unknown. Interestingly, metabolism alterations in transition metals, as iron, are known to be involved in the pathogenesis of tauopathies. It has been demonstrated that iron resides in tau tangles in postmortem brains of AD patients [118]. Moreover, the binding of iron to tau induces its conformational changes facilitating tau aggregation [119]. It has also been reported that iron can influence the pathways linked to tau hyperphosphorylation by regulating the activity of GSK3β and CDK5 [120]. If on the one hand not much is known about the causes of iron accumulation in tauopathies, on the other hand it has been demonstrated that alterations in tau functions can impact on ferroportin-mediated iron export pathway. Indeed, Ferroportin 1 is stabilized at the cellular membrane by APP, which itself is transported to the neuronal surface by tau [121]. Furthermore, genetic inactivation of tau in mice (tau^-/-^) blocks the trafficking of APP leading to brain iron accumulation [122]. Consequently, the increase of iron in a dysregulated system, can cause an increase of labile iron pool (LIP) leading to the generation of reactive oxygen species (ROS) which, inevitably, lead to oxidative damage and neuronal death. Indeed, a high incidence of oxidative damage has been demonstrated in AD [123], and the interaction between iron and tau has been shown to act as a source of ROS in neurons [124].

APP is a transmembrane protein expressed in the CNS involved in important brain function including development, memory and synaptic plasticity [125, 126]. The sequential cleavage of APP by secretory enzymes, β secretase (BACE) and γ secretase composed of Presenilins (PSs), generate Aβ peptide [127]. Firstly, the β-secretase cleave APP at the N terminus of the Aβ domain to produce the membrane-bound fragment C99 and the secreted APP ectodomain APPsβ. C99 is in turn cleaved by γ-secretase to generate the intracellular domain of APP (AICD) and Aβ. The cut made by y-secretase produces a different Aβ isoform of 38-43 amino acids [128]. After its release as a monomer, Aβ and especially the Aβ_42_ isoform self-assembles to form the Aβ oligomers and amyloid fibrils, which aggregate in the senile plaques into the brain contributing to the neuronal loss and neurodegeneration [128, 129].

The pathological mechanism of AD is complex and accumulating evidence has proved that extracellular Aβ deposition and intracellular accumulation of hyperphosphorylated tau remain the primary neuropathologic hallmarks for AD. However, a huge amount of discoveries show important pathological roles for other cellular and molecular processes including neuroinflammation [130], oxidative stress [131], apoptosis [132], autophagy defects [133] mitochondrial dysfunction [134] and metal dysregulation [135, 136]. Some of these events take place during Aβ deposition and tau hyperphosphorylation [135].

### 4.1 Brain Iron dyshomeostasis in early, symptomatic AD patients and *post-mortem* brains

It dates back to 1953 the description of increased iron levels in AD patients’ brains [137]. Since, the link between both Aβ plaques and tau tangles formation with iron accumulation has been studied [138, 139]. However, the mechanism which triggers iron accumulation in the brain is not yet clear. In the paragraph below we report an updated collection of the preclinical studies which attempt to decipher it. Focusing on human studies, different trials conducted in patients with early cognitive decline i.e. mild cognitive impairment (MCI) and AD patients clearly show that iron dyshomeostasis is a phenomenon associated to neurodegeneration.

A study involving a total of 90 participants including 30 MCI, 30 AD and 30 controls showed by susceptibility weighted imaging (SWI) an increase in brain iron content already in MCI subjects compared to controls [140]. Indeed, in the pre-symptomatic phase of the disease, accumulations of iron have been detected in the same regions affected by amyloid pathology: hippocampus and cerebral cortex [118]. Moreover, in a recent retrospective study were enrolled 73 subjects with normal cognition, 158 MCI and 48 AD patients, by using QSM it has been shown the presence of iron in the cortex, in the cingulate and insular cortex of MCI and AD patients compared to controls [141]. With the same approach, other studies also showed the increase of iron levels in the putamen of MCI subjects [142]. Moreover, a study using Quantitative Susceptibility Mapping (QSM) MRI at 7 Tesla and 11-Carbon Pittsburgh-Compound-B PET showed a strong association between high brain iron levels and Aβ plaques in MCI subjects, associating this effect with an increased risk for AD-dementia [143, 144]. It has been suggested that iron accumulation combined with Aβ plaques deposition can accelerate the clinical progression of the disease and could be used as a marker for diagnosis [144]. These data clearly indicate that alterations of brain iron levels occur in the initial phase of neurodegeneration and could represent an early indicator of cognitive decline associated with dementia.

If on one hand soluble Aβ can bind the ferric form (Fe^3+^) of iron to avoid iron overload, this interaction is so strong that once it occurs, it cannot be dissociated and it could contribute to the production of ferrous (Fe^2+^) iron leading to the production of potent oxidant (ROS), that accelerates Aß deposition [145]. A study conducted in AD patients compared with healthy controls revealed by quantitative susceptibility mapping-MRI higher iron amount in the deep grey matter and neocortical region of AD patients [146]. A meta analysis study of 300 AD cases [147] showed iron accumulation in cortical areas; in *post-mortem* AD brains iron accumulation was found in the inferior temporal cortex [148].

Indeed, in *post-mortem* AD brains there is plenty of evidence of increased iron levels and association to amyloid and tau aggregation; the use of Synchrotron X-ray spectromicroscopy technology localized the presence of ferrous iron within amyloid plaques’ cores [149, 150], and also in association with cortical tau aggregation [151]. All together, we can conclude that high levels of iron in all these brain regions are detected already in MCI subjects and that are associated with the motor and cognitive decline typical of AD pathology, suggesting iron dyshomeostasis as a pathological feature of AD.

### 4.2 Molecular mechanisms behind iron dyshomeostais in AD

Iron dyshomeostasis described in human patients is largely studied in preclinical models of AD [138, 152] in order to decipher the mechanisms behind iron accumulation.

In physiological condition APP is processed by α secretory enzyme to produce non-toxic forms of P3, Aβ16 and Aβ17-40/42; on the downside, the increase of iron levels in cells activate the amyloidogenic pathway promoting APP cleavage by β and γ secretases to form Aβ1-40 and Aβ1-42 fragments, inducing cells damage and death [149]. Indeed, the presence of the iron-responsive element (IRE) in 5’UTR of APP mRNA, closely linked to intracellular iron amount [153, 154], adds important mechanistic insights suggesting a role for iron in the regulation of AD-related genes transcription and in AD pathology. Interestingly, iron levels can modulate also the translation of APP through the regulation of the IRP binding to IRE in the 5′-UTR of APP mRNA, following the same mechanism of iron regulatory genes [153].

Because of this iron-mediated regulation of APP, when in condition of brain iron deficiency IRP1 can bind APP-IRE with high affinity repressing APP translation; the opposite occurs in iron overload conditions where APP translation is upregulated by virtue of IREs in the 5’UTR mRNA with the consequent increase in Aß deposits generated by enhanced APP processing [153].

Moreover, another direct link between APP and iron is the presence on the APP protein of a target sequence (or motif) that interacts with Fpn1 [155], and this can facilitate intracellular iron export stabilizing Fpn1 on human brain microvascular endothelial cells [156]. Furthermore, in a knockout mouse model of APP [157] and in patients with the Italian mutation on APP (A673V) [158] the silencing/mutation of APP leads to abnormal Fpn1 function resulting in inefficiency of neurons to export iron, with consequent iron accumulation, revealing that APP/Fpn1 plays an important role in the modulation of iron homeostasis in the brain [157]. In parallel, it is widely accepted that in the context of aging, the age-dependent Fpn1 is downregulated [4], and also in the APPswe/PS1dE9 mouse model and in brain tissues of AD patients [72]. Moreover, in the cohort of AD patients, the decrease in Fpn1 levels is accompanied by cognitive impairment [72]. To corroborate this hypothesis Bao and colleagues demonstrated that deficiency of Fpn1 induce brain atrophy, a distinctive pathological sign in AD.

A recent study showed that the binding of APP to β-secretase is induced by iron accumulation promoting Aβ accumulation and reducing the affinity of APP/Fpn1 and the consequence iron export from microglia cells [159]. This is also closely related to what has been described in both AD patients and animal models in which Fpn1 is downregulated, resulting in intracellular iron retention [160]. Since Cp is fundamental for iron oxidation before export, functional alterations in Cp have been found in AD as the event leading to iron retention [161, 162]. It is also noteworthy that the export of iron from neurons is closely related to the presence on the cell membrane of the metalloprotein APP, which by stabilizing Ferroportin 1 facilitates the export of iron contrary to what happens in APP knockdown models and in neuroblastoma SH-SY5Y cells, resulting in neuronal iron overload [163, 164].

Related to Fpn1 and to the regulation of brain iron metabolism, the hormone Hepcidin (Hepc) covers an important role being expressed in cortical neurons, glial cells and in the brain microvascular endothelial cells (BMECs) [44]; moreover, Hepc can bind Fpn1 in the cell membrane of BMECs promoting Fpn1 internalization and degradation blocking the iron passage through the BBB into the brain [165]. In the context of AD different studies have demonstrated that Hepc expression levels decrease in AD human and in the APP-transgenic mouse [160], while the Hepc treatment on cultured microvascular endothelial cells and neurons generated a reduction of iron import (TfR1 and DMT1) and export (Fpn1) proteins with a consequence reduction of iron intake in neurons [166]. Moreover, in a recent study conducted on APP/PS1 mice model it has been demonstrated the Hepc overexpression in astrocytes provokes a reduction in iron levels in cortical and hippocampal neurons with a significant improvement in terms of cognitive decline and Aβ plaques aggregation. These lead to a reduction of oxidative stress and neuroinflammation and more importantly, a decreased neurons death [167]. Although studies of Hepc in brain iron regulation need to be further developed, Hepc potential protective role in brain iron regulation is becoming more evident and starts to be recognized by the scientific community.

### 4.3 Molecular mechanisms of Ferroptosis

The balance of physiological processes is finely tuned by programmed cell death, but its deregulation contributes to the onset of various disorders. The main process of controlled cell death is apoptosis together with necrosis and autophagy [168]. However, in AD new kind of neuron loss have been described: necroptosis [169] and ferroptosis [170]. The latter is an iron-dependent, non-apoptotic and oxidative cell death type that was first described in 2012 by Dixon.

When iron levels increase, there is enhanced susceptibility to cellular death because of increased toxicity due to the accumulation of the metal within the nervous tissue. Indeed, since iron is an essential element of the catalytic subunit of lipoxygenase (LOX), involved in the oxygenation of polyunsaturated fatty acid [171], iron accumulation represents a crucial event in the generation of ROS promoting cellular death [172]. As the name suggests: “ferroptosis” from the Greek “ptosis”, meaning “fall” and Ferrum or iron, iron is involved as a distinctive peculiarity of this phenomenon of cell death. Ferroptosis gets involved in the onset and progression of lung, pancreas and gastrointestinal tumors, nervous system diseases (including NDDs, stroke and traumatic brain injury), ischemia-reperfusion in the liver, spleen and kidney injury [8, 173]. Biochemically, lipid peroxides cannot be metabolized, due to reduction of glutathione peroxidase 4 (GPX4) activity and intracellular glutathione depletion (GSH). Consequently, iron (Fe^2+^) can oxidize lipids resulting in reactive oxygen species (ROS) accumulation, accompanied by a dysregulation of mitochondrial structure/function, triggering ferroptosis [174].

Indeed, the morphological changes at the cellular level due to ferroptosis are: *i*) cell swelling, *ii*) reduced or absent mitochondrial cristae, *iii*) decreased mitochondrial membrane density that leads mitochondrial and cellular membrane rupture [175].

By the Fenton reaction, the increase of iron in the cell supports lipid peroxidation and ROS production which triggers ferroptosis. The association between iron gene/metabolism and ferroptosis activation has been amply demonstrated. As matter of fact, TFRC silencing can inhibit erastin-induced ferroptosis, preventing labile iron pool (LIP) accumulation [176]. Moreover, silencing of iron-responsive element-binding protein2 (IREB2) through shRNA causes the alteration of FtH, FtL and TfR1 expression, modifying iron intake and storage [170].

Inside the cells, ferritin, the main iron storage protein, can be degraded through different ways: in the lysosome under iron chelation stimulus (Deferasirox) [177], in the proteosomal pathway induced by overexpression of Fpn1 [178], or through an evolutionarily conserved degradation pathway named “ferritinophagy” that involves the protein Nuclear receptor Coactivator 4 (NCOA4) [67, 177]. This represents an important trigger in ferroptosis.

Since the interaction between NCOA4 and ferritin is FtH specific during ferritinophagy [67], NCOA4 favors FtH-rich ferritin heteropolymers degradation over FtL polymers as demonstrated the rate of iron release in different tissues and organs. Moreover, data on transgenic mice expressing FtH from a tetracycline-inducible promoter showed that FtH expression can change according to iron amount, inducing an iron deficiency phenotype. This means that FtH regulates tissue iron balance.

### 4.4 Ferroptosis in AD

**Figure 2.**
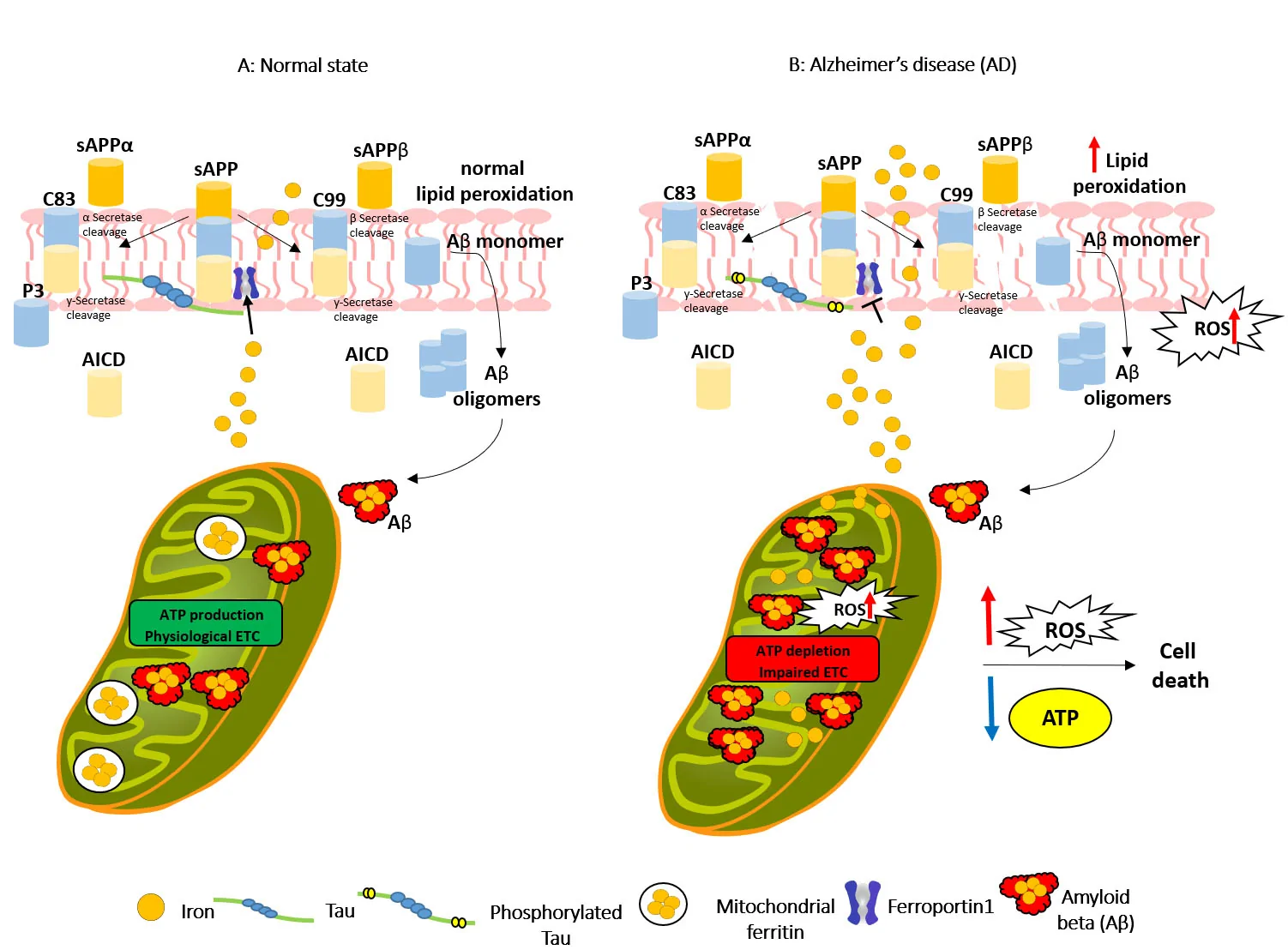
**Mitochondrial Iron dysregulation in Alzheimer’s disease**. (**A**) Normal state: physiologically, the amyloid precursor protein (APP) can stabilize Ferroportin-1 (Fpn1) to promote iron export and Tau protein supports APP transport to the cell surface. Aβ is produced in soluble form during normal cellular metabolism [263]. (**B**) In AD: Tau is hyper phosphorylated and aggregated resulting in a decrease in Tau, needed for APP transport. This leads to APP cleavage and Aβ generation, with a consequence alteration of iron efflux through Fpn1, generating iron accumulation in cells. Moreover, the binding of iron to Aβ accelerates its aggregation, leading to alteration in mitochondrial membrane permeability and reactive oxygen species (ROS) production inside the mitochondria and iron accumulation in mitochondrial ferritin. Related to this, Aβ overproduction damages mitochondria causing dysfunction of mitochondrial complexes activity, leading to ROS production and adenosine triphosphate (ATP) depletion. ROS overproduction also induces membrane damage due to lipid peroxidation and triggers cell death. Inkscape software (Free vector graphics editors) was used to create the image.

In the context of neurological diseases, ferroptosis covers an important role [179] in NDDs including Parkinson’s and Alzheimer’s diseases related to a potential mechanism of neuronal loss [72, 180]. The main markers of ferroptosis, iron dyshomeostasis, lipid peroxidation and ROS production have long been identified both in the brain of AD patients and in animal models [181]. Regarding iron metabolism pathway, even though the specific mechanism of iron in ferroptosis is not clear yet, its involvement in terms of iron dyshomeostasis is certainly proven. Moreover, the main features related to ferroptosis observed were the expression of ferroptosis’ markers, ACSF2 and IREB2 upregulation accompanied by a downregulation of GPX4 [72]. Moreover, Fpn1 restoration and *in vitro* inhibition of ferroptosis ameliorated the neuronal death and memory impairment induced by Aβ1-42 [72]. This could mean that Fpn1 downregulation not only plays a role in the induction of AD but also of ferroptosis in the pathogenesis of AD (Fig. 2).

It has been accepted that lipid peroxidation products drive ferroptosis and occurs preferential on polyunsaturated fatty acids (PUFAs) [182] present at high levels in the brain where it ameliorate the fluidity and plasticity of the membrane in order to allow the release of neurotransmitters and neuronal network development [183, 184]. The process of lipid peroxidation results to be high in the brain of AD and beyond is considered an early event in the pathogenesis of AD [185]. The co-localization of lipid peroxidation products with Aβ it was discovered by Butterfield and colleagues; they demonstrated that Aβ peptides led to lipid peroxidation (indexed by HNE) in AD brains [186] with a consequence enhancer of APP processing [187].

Moreover, it was found in AD a change in several classes of lipid peroxidation involved in PUFA deoxygenation [188, 189]. In particular, it was found an increase of the enzymatic activity of 12/15-lipoxygenase (12/15-LOX) in AD patients, and it is accompanied by an increase of its protein amount in Triple transgenic (3xTg) that has negative effects on memory and learning capacity.

*In vivo* treatment with PD146176, inhibitor of 12/15-LOX, reverse the phenotype with a strongly decrease of Aβ and tau levels and aggregation and an increase of synaptic integrity [190]. Moreover, in a study conducted on APP/PS1 double mutant transgenic mice, dietary deuterated PUFAs, which can reduce membrane lipid peroxidation, reduced the concentration of lipid peroxidation products as well as Aβ without shown any sign of spatial learning and memory impairment [191]. Many others fatty acid inhibitors, as well as Sphk1 and 2 [192], CMS121 [193] exert mechanisms aimed at the protection from ferroptosis alleviating or improving AD-like pathology both in patients and mice.

Another vulnerability factor associated to ferroptosis in AD is the inhibition of Gpx4, an antioxidant enzyme expressed in the brain. Studies have proven that Gpx4 is a crucial regulatory enzyme in ferroptosis-related to AD [194, 195].

In particular, in a Gpx4BIKO conditional knockout mice model, the deletion of Gpx4 in hippocampal and cortical neurons, caused cognitive impairment in terms of memory and learning, with a marked hippocampal neurodegeneration, increase of lipid peroxidation and neuroinflammation [195] all markers associated with ferroptosis. In the same study, while the Gpx4BIKO fed with a Vitamin E deficiency diet increased hippocampal neurodegeneration and cognitive impairment, the treatment with a ferroptosis inhibitor seemed to improve neurodegeneration [195].

Therefore, the significant dysregulation of iron metabolism, perturbation of lipid metabolism and redox homeostasis suggested an early involvement of this pathway in AD pathology.

Indeed, it is interesting to note that, in the brain of AD patients and the *in vitro* model of IMR-32 cells, the expression of mitochondrial ferritin (FtMt) was upregulated playing an important antioxidant role [196]. Conversely, in FtMt knockout mice injected with Aβ25-35, the Aβ-induced neurotoxicity was enhanced [197].

Indeed, energy alteration is one of the signs of neurodegeneration in AD and is mostly attributable to impaired mitochondrial functions. In the context of neurodegeneration, such as AD, mitochondrial dysfunction occurs with decreased ATP production, increased ROS production and neuronal loss. Indeed, the alteration of DRP1 activity is correlated to an increase of mitochondrial fragmentation implicated in AD [198, 199], but it is still unknown the mechanism by which iron induces mitochondrial alteration in AD. Since the oxidative stress is linked to Aβ production [200, 201], it is plausible that there is a correlation with the alterations of mitochondria functions and morphology. Indeed, in the early stage of AD, it has been identified a decreased ATP production in the brain [202]. In mitochondria, the presence of an active γ-secretase complex (involved in Aβ production), and of APP protein, can affect OMM and, in turn, can impair protein shuttle import. Moreover, Aβ is able to inhibit complex IV of respiratory chain, and to bind Aβ-bound alcohol dehydrogenase, which is involved in the production of ROS [203]. In addition, the Aβ-induced toxicity is a putative cause of both mitochondrial dysfunction and apoptosis [204].

Altogether, mitochondria are in the same time the source and target of toxic ROS: therefore, mitochondrial dysfunction and oxidative stress are crucial in age-related NDDs, as well as AD [134].

## 5. Iron modulators: targeting iron as a potential therapeutic avenue to control AD course

To date, the pharmacological options developed for AD are not curative but able to relieve the symptoms of the disease (*i.e.* β-secretase converting enzyme inhibitors) [205]. Recently, two anti-amyloid-β monoclonal antibodies, the humanized IgG1 monoclonal aducanumab and lecanemab have been approved by the FDA [206]. Both immunotherapies are promising in reducing brain amyloid levels and cognitive decline when applied to early AD patients; further studies in terms of safety and efficacy are ongoing [206]. However, different evidence suggests that the use of anti-amyloid-β therapy in the symptomatic phase of AD is a too delayed treatment. Other important aspects, not to be underestimated, are related to the presence of *i)* BBB, which can obstruct drug passage and can reduce the amount of drug reaching the brain, *ii)* reduction of its bioavailability due to first-pass metabolism, *iii)* the way of drugs administration but also *iv)* extensive side-effects due to site non-specificity and *v)* drugs toxicity [207].

It is necessary to investigate new aspects/mechanisms of neurodegeneration in AD using new drugs or existing compounds approved for other medical indications that can modify or prevent the disease. In this context, considering the amount of evidence highlighting the role of iron dyshomeostasis and accumulation in neurodegeneration, iron could represent a new interesting target for therapeutic approaches for AD. The process of drug repurposing can represent an innovative approach to find promising options for AD. In this perspective, although in clinical practice iron chelators are used in patients with systemic overload disease (hemochromatosis), beta thalassemia major, sickle cell anemia, myelodysplasia, and aplastic anemia [208], several studies showed that many iron chelators compounds (Table 1) have potential effect for AD treatment. Iron chelators could be used also in combination with other treatments that are more canonical and already know to potentially have minor but beneficial effects such as antioxidants, acetylcholinesterase and ferroptosis inhibitors. These compounds are also discussed below.

### 5.1 Iron chelators

Iron chelation is a complex process: a chelator should be able to pass the BBB and to trap iron specifically in the regions where iron overload occurs, without depleting transferrin bound iron from the plasma and to transfer it to other proteins such as circulating transferrin.

Three iron chelators have been approved up to now by Federal Drug Administration (FDA) for the symptomatic treatment of NDs: Deferoxamine, Deferasirox (Exjade) and Deferiprone. But, while Deferoxamine and Deferasirox do not easily cross the BBB and bind iron in a dose dependent manner, the molecular structure of Deferiprone allows to cross the BBB and it is the drug of choice in the majority of clinical trials for neurodegenerative disorders [209-211].

Analyzing the three drugs more closely, the therapeutic effects of Deferoxamine are promising in the treatment of AD; this compound seems to act especially on extracellular iron [212] in a 1:1 ratio [213, 214] and can modulate the expression of different genes (hypoxia-inducing factor, IRP-1, and APP) with a blocking effect on ROS production [215].

Indeed, an *in vivo* study using APP/PS1 transgenic mice fed with iron-enriched diet showed that after Deferoxamine injection, iron-dependent tau phosphorylation is inhibited *via* the CDK5 and GSK-3β pathways [216]. More in details, CDK5 activity is decreased by Deferoxamine and, as consequence, GSK-3β is phosphorylated and inactivated, resulting in a decrease of tau phosphorylation. Moreover, Deferoxamine injection in APP/PS1 transgenic mice inhibits the formation of amyloid-β and it improves memory deficits [215].

Crapper and colleagues reported in a phase II clinical trials where intramuscular Deferoxamine administration reduces the cognitive decline in AD patients [217]. It was also demonstrated that Deferoxamine, as well as Deferiprone, ameliorates BBB integrity, reducing brain iron overload and brain mitochondrial alterations [211].

However, although Deferoxamine has been approved by FDA, the clinical application is still tricky due to *i*) its poor bioavailability; *ii*) low ability to cross the BBB; *iii*) and its way of administration through injection; it represents one of the few clinical studies of drug repositioning which is potentially capable of modifying AD disease in terms of cognitive improvement.

**Table 1:** **List of preclinical and clinical trials testing iron chelators compounds as potential treatments for AD**. Compounds are grouped based on the therapeutic function. The drug/molecule is indicated together with the route of administration, the capability or not to pass the BBB and references to preclinical or clinical trials. N/A stands for not available.

Therapeutic Function	Drug/Molecule	Administration	BBB passage	Preclinical Studies	Preclinical Results	Clinical Trials	Clinical Results
**Iron chelators**	Deferoxamine	Intramuscular/ Subcutaneous	Low	*In vivo*	*In vivo*: Tau phosphorylation, APP inhibition; improve memory deficits [215, 216].	Phase II [217].	Reduces the cognitive decline [217].
	Deferasirox	Oral	Limited	*In vitro*/*vivo*	*In vitro*: apoptosis inhibition [219]. *In vivo:* mitigates learning and memory deficits [219];reduction in tau hyperphosphorylation [220].	N/A	N/A
	Deferiprone	Oral	Optimal	*In vitro*/*vivo*	*In vitro*: abrogated neuronal cell death [222]. *In vivo*: mitigate BBB damage, alteration in mitochondrial dynamics, the increases of tau-hyperphosphorylation and Aβ accumulation and reduction of dendritic spine [211].	Phase II ClinicalTrials.gov ID NCT03234686	Underway
	M30	Oral	Optimal	*In vitro*/*vivo*	*In vitro*: reduction of β-amyloid plaques SH-SY5Y and APP-CHO cells [225]. *In vivo*: reducing in brain iron accompanied by a significantly reducing in brain deposition of Aβ-40 and Aβ-42 and Aβ plaques; improve improves cognitive impairments [224]	N/A	N/A
	Hepcidin	Intraperitoneal	N/A	*In vivo*	*In vivo*: in APP/PS1 double-transgenic mice injected with pAAV-gfap:Hamp, induced a reduction of cognitive decline accompanied, Aβ plaques formation, iron levels, oxidative stress, neuroinflammation and neuronal death, [150].	N/A	N/A

Regarding the oral iron chelator Deferasirox (Exjade), contrary to Deferoxamine, it acts on intracellular iron in a 2:1 ratio [213, 218] and its ability to pass the BBB is improved if in combination with lactoferrin (Lf) [219]. The use of Lt conjugates with Deferasirox displayed a neuroprotective effect both *in vitro* model of PC12 neuronal cell in term of apoptosis inhibition but also *in vivo* rat model of AD [219]. Interestingly, the intraperitoneal administration of the conjugates *in vivo* mitigates learning and memory deficits induced by amyloid pathology [219]. In a recent work the comparison between three different AD mice model, APP (Tg2576), Tau/Tau (JNPL3) and APP/Tau (Tg2576/ JNPL3), after Deferasirox administration showed that although the treatment did not affect memory and motor functions, but it revealed a reduction in tau hyperphosphorylation. The authors hypothesized that the drug may act by chelating iron that is the driving force on the tau aggregation or it could directly bind tau in order to prevent its aggregation [220].

Deferiprone is another promising oral iron chelator able to binds intracellular iron [212] in a 3:1 ratio [213, 221] and unlike the other two chelators it is able to readily cross the BBB [212]. The demonstration that also Deferiprone exhibits neuroprotective effect is attributable to a study conducted on primary neuronal cultures treated with ferric (Fe^3+^) iron, human Aβ1-40 and Aβ1-42. The treatment with the drug at different concentrations abrogated neuronal cell death [222], supporting also the hypothesis of the central role of iron in Aβ induced neurotoxicity [139]. To corroborate this hypothesis an *in vivo* study conducted on rats fed with an iron-rich diet demonstrated that iron overload can promote BBB damage causing the increase of iron in the brain, alteration in mitochondrial dynamics, and the increases of the two markers of amyloid pathology: tau-hyperphosphorylation and Aβ accumulation and finally the strongly reduction of dendritic spine. The treatment with Deferasirox or Deferiprone mitigates these damages [211].

As for Deferiprone, a clinical study appears to be underway on the use of this drug in Alzheimer's disease (Deferiprone to Delay Dementia (The 3D Study) ClinicalTrials.gov ID NCT03234686). This is a phase 2, randomized, placebo-controlled, multicenter study focused on *i*) evaluating the safety and efficacy of the drug and whether *ii*) it slows cognitive decline in patients with prodromal AD (pAD) and Mild AD (mAD). Some new iron chelators, that have been tested both *in vivo* and *in vitro* in AD models, showed positive effects in terms of reduction of APP expression and Aβ deposition. Here we reported some crucial information about their mechanism of action. M30, an oral iron chelator compound, is able to cross the BBB and it is considered a multimodal drugs with double function: not only it acts as an iron chelator but also as inhibitor of monoamine oxidase (MAO)-A and -B [223]. The pharmacological effect of M30 is broad spectrum including neurological effects in terms of rescue in neuronal death and differentiation. In the context of AD, the administration of M30 in APP/PS1 Tg mice model showed reducing in brain iron accompanied by a significantly reducing in brain deposition of Aβ-40 and Aβ-42 and Aβ plaques [224]. Moreover, from a behavioural point of view, M30 treatment improves cognitive impairments as memory, learning capacity and anxiety [224]. All these findings are in line with *in vitro* studies on SH-SY5Y cells line and Chinese hamster ovary cells (CHO) transfected with the APP with the human Swedish mutation [225]. Interestingly, M30 administration induced a reduction of β-amyloid plaques in the principal areas affected by the pathology probably due to a decreasing of APP levels through 5′UTR of the APP transcript [226]. An emergent class of new chelators 2-amido-3-hydroxypyridin-4-one seem to be neuro-protective against the neurotoxicity induced by β-amyloid aggregation. *In vitro* results on cortical neurons treated with iron (FeNTA) or amyloid-β 1-40 (Aβ1-40) shown as the iron chelator treatment, in a dose dependent manner, reversed the neuronal death induced by iron [227]. Last but not least, it is worth considering also the use of Hepcidin, the iron regulatory hormone, in AD treatment. Indeed, several evidence have demonstrated that Hepcidin can reduce iron influx by preventing iron overload in the CNS. In particular a study demonstrated that APP/PS1 double-transgenic mice injected with pAAV-gfap:Hamp, an astrocyte-specific Hepcidin expression Adeno-Associated Virus [228], showed a reduction of cognitive decline accompanied by a moderate reduction of Aβ plaques formation, iron levels, oxidative stress, neuroinflammation and neuronal death, in the two main regions affected by the pathology, cerebral cortex and hippocampus [167]. This protective effect is achieved by the fact that Hamp overexpression in astrocytes at the level of the BBB influences iron income in the brain and, at the cellular level, this impacts also in the astrocytes/ neuron’s crosstalk. Indeed, Hamp overexpression in astrocytes determines an increase of Hepcidin secretion resulting in a reduction of iron export and deposition in neurons. Consistently, FPN1 degradation in BMVECs is accompanied by a reduction of iron levels in neurons of APP/PS1 double-transgenic mice injected with pAAV-gfap:Hamp [167]. However, it is to note that, the route for Hepcidin administration in the brain and its potential side-effects need in-depth studies and further investigations before moving from preclinical to clinical studies.

### 5.2 Combinatorial/second hand approaches: antioxidants, acetylcholinesterase and ferroptosis inhibitors

To have an overview of drugs that have been tested in AD, we report below a list of compounds classified as antioxidants, acetylcholinesterase and ferroptosis inhibitors which exert benefits by acting on different targets or which combined with other compounds, could exert beneficial effects in terms of cytoprotection, neuroprotection and promising in the treatment of AD (Table 2).

**Table 2:** **List of compounds acting as antioxidants, acetylcholinesterase and ferroptosis inhibitors**. Compounds are grouped based on the therapeutic function. The drug/molecule is indicated together with the route of administration, the capability or not to pass the BBB and references to preclinical or clinical trials. N/A stands for not available.

Therapeutic Function	Drug/ Molecule	Administration	BBB passage	Preclinical Studies	Preclinical Results	Clinical Trials	Clinical Results
**Antioxidants**	Clioquinol (CQ)	Oral	N/A	*In vitro*/*vivo*	*In vitro:* can reduce secreted Aβ levels in cell culture by inducing metal-dependent activation of PI3K and JNK [232]. *In vivo*: reduced cerebral Aβ deposition without any adverse effects [233].	Phase II [235].	Decrease on Aβ42 in CSF and an increase on Zinc levels in the plasma prevented cognition deterioration without any side effects [235].
**Acetylcholinesterase inhibitors**	HLA20A	N/A	N/A	*In vitro*	*In vitro*: reversed the neuronal death induced by iron HLA20A can inhibits AChE promoting the release of the chelator active form, HLA20 capable of inducing neuroprotection. Reducing the expression of both APP and β-amyloid aggregation [237].	N/A	N/A
	Coumarin	N/A	N/A	*In vitro*/*vivo*	*In vitro*: cytoprotective effects in glioma cell line (U251) [240]; *In vivo:*improve cognitive alteration in scopolamine-induced AD mice [240].	N/A	N/A
**Ferroptosis Inhibitors and Antioxidants**	Vitamin E	Oral	N/A	*In vivo*	*In vivo*: increase in hippocampal neurodegeneration and cognitive dysfunction in Gpx4BIKO mice treated withVitamin E deficient diet[195].	N/A	Treatment of patients with mild-moderate AD showed reduction in cognitive decline and caregiver burden [241]; The treatment in patients with mild cognitive impairment (MCI) or AD prevent dementia progression and improve cognition [242].
	Liproxstatin1 (Lip-1) or Ferrostatin1 (Fer-1)	N/A	N/A	*In vitro*/*vivo*	*In vitro:*in primary hippocampal neurons of mice induced by Aβ aggregation, revealed effectively reducing of neuronal death and memory impairments [72].	N/A	N/A
	α-Lipoic acid (LA)	N/A	Optimal	*In vivo*	*In vivo*: the LA treatment in different in vivo models improved cognitive alteration and memory. [249, 250] [251]. Moreover, in a triple transgenic mice of AD restore both glucose metabolism and synaptic plasticity [252, 253].		Moderate cognitive impairment in AD patients [247, 248].
	Selenium (Se)	Oral	N/A	*In vitro*	*In vitro:*in SH-SY5Y neuroblastoma cells line expressing Swedish APP mutant Se can reduce Aβ production by reducing β-secretase and y-secretase activities preventing the toxicity mediated by Aβ [260].	Pylot Trial	A pilot study of 40 AD cases showed that after the oral Se revealed the Mini-Mental Status Examination (MMSE) score did not deteriorate [261].
	Mito Q		N/A	*In vitro* and *in vivo*	*In vitro:* in cortical neurons and in MitoQ attenuated β-amyloid (Aβ)-induced neurotoxicity in cortical neurons and also prevented increased production of reactive species and loss of mitochondrial membrane potential (Δψ(m). *In vivo:* in a triple transgenic mouse model of AD (3xTg-AD), MitoQ injection prevents cognitive decline, oxidative stress, β-amyloid accumulation, astrogliosis, synaptic loss in the animals’ brains [262]	N/A	N/A

Clioquinol (CQ) is an antibiotic drug with moderate ability to chelate iron (Fe), copper (Cu) and zinc (Zn), termed [229, 230]. One study showed that CQ can chelate ions, leading to the breakdown of Aβ aggregates although it has not been shown that it can simultaneously prevents the progression of Aβ aggregation [231]. *In vitro* study on APP-transfected Chinese hamster ovary cells (CHO) treated with Fe, Cu or Zn showed that the CQ treatment can reduce secreted Aβ levels in cell culture through the activation of phosphoi-nositol 3-kinase and JNK [232]. In addition, *in vivo* study on Tg2576 APP transgenic mice, the oral CQ treatment (20 mg/kg daily for 9 weeks) reduced cerebral Aβ deposition without any adverse effects [233]. Despite these positive results, the use of the CQ was discontinued because it seemed to be involved in subacute myelo-optico-neuropathy (SMON), probably due to the deficiency of vitamin B12 [234]. However, in a pilot study of phase II conducted in AD patients revealed that the group of patients treated with CQ showed a decrease on Aβ42 in Cerebrospinal fluid (CSF) and an increase on zinc levels in the plasma compared to placebo one, improving also cognition without any side effects [235].

A combinatorial activity between acetyl-cholinesterase (AChE) inhibition and iron chelation is given by HLA20A. The development of this new drug is related to the discovery of the co-localization of AChE with beta amyloid which can accelerates its aggregation [236]. HLA20A can inhibits AChE promoting the release of the chelator active form, HLA20 capable of inducing neuroprotection [237]. The capability of the drug to induce neuroprotection was correlated with a reduced expression of both APP and β-amyloid aggregation induced by iron, copper and zinc [238].

Finally, Coumarin and its derivatives seem to work as potential agents in the treatment of AD [239] acting both as acetylcholinesteresis (AChE) inhibition and iron chelators [239]. Moreover, a mixed compound composed by hydroxypyridinone and Coumarin seems to have a double function: iron chelation and MAO-B inhibition. This new double target can be promising in AD treatment since this novel compound showed cytoprotective effects in glioma cell line (U251) and significant improve in cognitive alteration in scopolamine-induced AD mice [240].

It is now known that ferroptosis covers an important role in the pathological process of AD suggesting that this process of programmed death, can be considered a potential therapeutic target for AD treatment. Especially, the use of ferroptosis inhibitors seems to have beneficial effects in clinical trials. Focusing on the ions chelators Desferrioxamine (DFE), in a single-blind study, the intramuscular injection on 48 patients affected by a probable AD compared to oral placebo (lecithin), or no treatment revealed that although there were no differences in the rate of deterioration or in the measures of intelligence, memory, or speech ability the DFE treatment reduced the rate of cognitive impairment [217]. This study reveals how the use of DFE can reduce the progress of dementia in AD patients.

Also, a promising clinical trial showed that Vitamin E, despite its little anti-ferroptotic potential, appears to have beneficial effects in the treatment of patients with mild-moderate AD in term of reduction in cognitive decline and caregiver burden [241]. In another randomized clinical trial Vitamin E administration gave positive results in terms of preventing the progression of dementia and, in the same time, by improving cognition in patients with mild cognitive impairment (MCI) or AD compared to placebo, without any signs of severe side effects [242].

Moreover, since Glutathione peroxidase 4 (Gpx4) is a key regulator of ferroptosis, *in vivo* study on forebrain neuron specific Gpx4 knockout mice (Gpx4BIKO) it was demonstrated that the treatment with Vitamin E deficient diet caused an increase in hippocampal neurodegeneration and cognitive dysfunction [195].

In order to understand if ferroptosis takes place in neuronal death and cognitive decline in AD, recently two ferroptosis inhibitors, Liproxstatin1 (Lip-1) or Ferrostatin1 (Fer-1), have been tested. The administration of these two ferroptosis inhibitors in primary hippocampal neurons of mice induced by Aβ aggregation, revealed effectively reducing of neuronal death [72].

Even though these studies proved the efficiency of Fer-1 in improving oxidative stress and preventing ferroptosis not only in AD but also in Huntington’s, Periventriculae leukomalacia, kidney dysfunction [243, 244], there are no clinical trials available.

Moreover, α-Lipoic acid (LA), an antioxidant and iron chelators compound, [245, 246] exhibit neuroprotective properties against AD and it can be effective in BBB crossing. The therapeutic effect of LA was demonstrated in clinical trials where it has been shown that it may moderate cognitive impairment in AD patients [247, 248]. LA treatment in different *in vivo* models such as Senescence Accelerated Mice (SAMP8), aged rats [249, 250] and in Tg2576 model of AD improved cognitive alteration and memory [251]. Moreover, in a triple transgenic mice of AD restore both glucose metabolism and synaptic plasticity [252, 253].

Based on these findings, it has been proposed that these mechanisms are related to anti-inflammatory [254], antioxidant [255] and antiamyloidogenic features of LA [256]. Although LA is considered a potential therapeutic compound for AD, the mechanism of action remains unknown especially in terms of its capacity in controlling tauopathy and neuronal death. In this context a recent study revealed that LA supplementation on P301S Tau transgenic mice can inhibit Tau hyperphosphorylation induced by iron overload and improvement in cognitive impairment. In the same study, the main markers of ferroptosis, such as inflammation and lipid peroxidation, are inhibited by LA supplementation [246]. All together these results are promising for the use of this compound in the tauopathies treatment since LA exhibited an important role in inhibiting Tau hyperphosphorylation, neuronal loss and ferroptosis.

Selenium (Se), is a trace element with antioxidant property able to modulate important brain functions [257], to reduce activity in the brain [258]. In fact, a decrease of Se amount in the brain altered the activity of the selenoenzyme as Glutathione Peroxidase (GPx) decreasing at the same time the antioxidant protection in the brain [259]. *In vivo*, *in vitro* and clinical trials studies in AD animal models and patients, revealed a protective role of Se in the pathology progression. In particular, Gwon et al demonstrated that in SH-SY5Y neuroblastoma cells line expressing Swedish APP mutant and on primary hippocampal and cortical neurons treatment of Se for 12 h can reduce Aβ production by reducing β-secretase and y-secretase activities preventing the toxicity mediated by Aβ [260]. A pilot study of 40 AD cases showed that after the oral Se administration the supranutritional selenium integration it was well tolerated and the responsive groups, in which the selenium concentration in CSF was increased, revealed that the Mini-Mental Status Examination (MMSE) score did not deteriorated [261]. Of course, more clinical and experimental studies are needed in order to provide a correct answer of the beneficial role of Selenium in the treatment of AD.

To conclude the overview of the use of antioxidants in AD treatment, it is important to mention a novel mitochondria-targeted antioxidant MitoQ (mitoquinone mesylate). An *in vivo* study demonstrated that MitoQ treatment in 3xTg-AD mice ameliorates cognition and prevents oxidative stress, β-amyloid accumulation, astrogliosis, synaptic loss in the animals’ brains [262].

## Conclusions and Perspectives

The mechanism driving iron dyshomeostasis and overload in neurodegenerative disorders is far from being described. However, it is evident from the data collected in the literature on human brains from MCI subjects, AD patients and *post-mortem* AD brains that brain iron dyshomeostasis and accumulation correlates with progressive neurodegeneration and cognitive decline.

Also, the studies conducted *in vitro* and *in vivo* highlight a clear role for iron dyshomeostasis and ferroptosis in models of neurodegenerative diseases, especially AD and attempt to decipher the molecular mechanisms behind iron accumulation. Finally, clinical studies in which iron is targeted, showed a potential beneficial effect in the control of amyloid and tau pathology.

Altogether, the data reported from the literature show that brain iron dyshomeostasis and ferroptosis represent important features of the neurodegenerative process going on in AD and could represent new targets for potential treatments to be reproposed for AD, but also for other neurodegenerative disorders characterized by iron dyshomeostasis.
